# Emergent Dynamics of Thymocyte Development and Lineage Determination

**DOI:** 10.1371/journal.pcbi.0030013

**Published:** 2007-01-26

**Authors:** Sol Efroni, David Harel, Irun R Cohen

**Affiliations:** 1 National Cancer Institute Center for Bioinformatics, National Institutes of Health, Rockville, Maryland, United States of America; 2 Department of Computer Science, Weizmann Institute of Science, Rehovot, Israel; 3 Department of Immunology, Weizmann Institute of Science, Rehovot, Israel; Scripps Gerontology Center, United States of America

## Abstract

Experiments have generated a plethora of data about the genes, molecules, and cells involved in thymocyte development. Here, we use a computer-driven simulation that uses data about thymocyte development to generate an integrated dynamic representation—a novel technology we have termed *reactive animation* (RA). RA reveals emergent properties in complex dynamic biological systems. We apply RA to thymocyte development by reproducing and extending the effects of known gene knockouts: CXCR4 and CCR9. RA simulation revealed a previously unidentified role of thymocyte competition for major histocompatability complex presentation. We now report that such competition is required for normal anatomical compartmentalization, can influence the rate of thymocyte velocities within chemokine gradients, and can account for the disproportion between single-positive CD4 and CD8 lineages developing from double-positive precursors.

## Author Summary

Biological systems are the embodiment of complexity that defies intuitive understanding. Biologists have accumulated masses of data about the molecules, cells, and discrete interactions that compose living systems, but the list of facts alone cannot explain how such systems work dynamically. We have developed a hybrid, computational approach to the simulation of complex systems called *reactive animation* (RA). RA uses a bottom-up integration of diverse experimental data to create an integrated and dynamic representation of the system's interacting cells and molecules. RA is faithful to experimental fact, while it plays out the action in animated formats directly accessible to the eye and mind. Most importantly, RA is analytical, interactive, and allows experimentation in silico. Here, we use RA to reveal unexpected emergent properties of thymocyte development. In particular, we now report that competition between thymocytes for sites of stimulation could be important in generating the fine anatomy of the thymus, in selecting for thymocytes with a range of migration velocities, and in explaining the paradox of CD4 to CD8 T cell lineage ratios. This study highlights the explanatory power and the potential aid to experimentation offered by an animated, interactive simulation of complex sets of data.

## Introduction

The mammalian thymus receives stem cells from the bone marrow. These cells—thymocytes—go through a series of anatomical subcompartments in a process termed *T cell education* [1,2]. About 97% of candidate T cells die, while the remaining 3% are essential to the continuing development of the adaptive immune system [3]. For a recent review of thymic architecture and cell traffic, see [4]. For a brief schematic animation of thymic maturation, see Video S1.

Extensive research in disparate disciplines has uncovered a mass of data regarding thymocyte development. Subfields of thymus research include genes, gene expression and differentiation; molecules (integrins, chemokines, cytokines, receptors, antigens, and other ligands); cells (stem cells, thymocytes, epithelial cells, dendritic cells, and macrophages); cell behavior (adhesion, migration, and anatomic localization); cell states (differentiation states, cell cycle, proliferation, and apoptosis); and physiology (antigen expression, positive and negative selection, lineage choice, and antigen-receptor repertoires). The technologies used to study thymopoeisis include genetics, transgenes and gene knockouts, protein chemistry, microscopy and immunohistochemistry, in vitro cell cultures and interactions, in vivo phenotypes, cell and organ transfers, immunizations, and more. A systematic integration of these data into an accurate and comprehensive representation is much needed. We address this need using reactive animation (RA) to reveal multiscalar emergent properties and to guide experimentation in thymocyte development.

**Figure 1:**
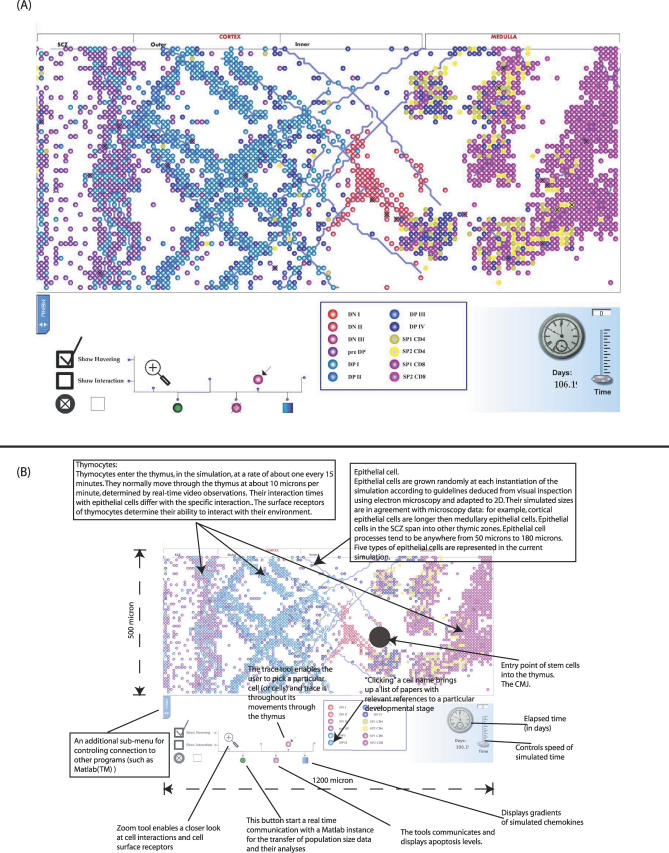
A Snapshot of the Simulated Thymus at Run Time (A) Different colors stand for different stages during thymocyte selection. The legend box shows the different developmental stages and their corresponding colors. (B) Different icons beneath the figure stand for different tools for the control and the visualization of the simulation. The tools include the ability to zoom into parts of the visualization, show and hide the two tiers of the simulation, highlight interacting cells, display or hide chemokine gradients, display statistical information through real-time communication with Matlab, visualize apoptotic levels in real-time, initiate and stop a backtrack of a cell's migration, and more. (A,B) In the lower right hand side, the elapsed time is displayed, together with a utility that enables the control of time progression.

RA is a computational approach to simulating complex dynamic systems. The technology of RA has been described elsewhere [5–7]. Briefly, the RA simulation is built in two tiers. The first tier is built, bottom-up, from the actual cellular and molecular data, and incorporates the program, the logic, and the dynamics of the simulation. The second tier is a front-end visualization of the simulation capable of real-time interactive manipulation of the action. RA allows the experimenter to extract statistical and local information from the running simulation. Moreover, the experimenter can intervene in the simulation and observe in silico the effects of thought experiments. Our simulation was built primarily using for the first tier the language of Statecharts [8], with its enhanced legibility and organizational structure. We added to Statecharts a second tier of animation. Between the two tiers, we built a set of tools to facilitate data-mining options, such as tracking cells, manipulation of surface receptors, inducement of apoptosis, tracking cell ancestry, data displays, visualization of chemokine gradients, zooming in and out across scales, streaming reports, statistical data, and more (see Figure 1B). Demonstrations of these tools are in Videos S2–S4 and online. The references to papers we used for the database are listed in Text S1.

RA differs fundamentally from other approaches directed to network modeling [9–11] or to simulating the microarchitecture of the immunological synapse or membrane [12–14]. We did not simulate membrane data in the present work due to the lack of quantitative data. Another spatially based system has been developed [15], and thus far it has been applied to molecular signaling [16].

## Results

### Thymus Anatomy Emerges from Molecular Data

Data at the level of single cells and their microenvironment culled from hundreds of papers were coded to the simulation. Anatomic localization is critical to thymus development; thymocytes at different developmental stages migrate to specific thymic compartments [2]. Validation of a bottom-up simulation such as RA (and of the database itself) requires that the microscale molecular data put into the model suffice to generate realistic macroscale thymocyte migration and anatomical location. Cell migration depends on thymocyte receptor profiles, chemokine gradients, epithelial cells, cell proliferation, cell survival, cell velocity, and other factors that enter the simulation. For example, a thymocyte at the double-negative (DN)1 stage expresses a profile of surface markers CD4^−^CD8^−^CD25^−^CD44^+^Lselectin^low^CD69^−^ [17]. Experimentally, we know that thymocytes at the DN1 stage migrate towards the chemokine CCL25 [18]. The thymus stroma, too, influences migratory decisions; a thymocyte's path to a chemokine may be blocked by another cell. Furthermore, the chemokine itself is involved in two dynamic processes: first, specific regions of the thymus continuously secrete the chemokine, and second, the chemokine diffuses over time and space. Thus, the migration of a thymocyte continuously changes as a function of secretion and diffusion of chemokines and the current locations of other migrating thymocytes and stationary stromal cells. This and much additional information is included in the simulation. Figure 1 demonstrates that the simulated thymic lobule faithfully produces the fine anatomical relationships of real thymic structure; thousands of thymocytes, individually computed, localize, as seen in histological sections, to particular anatomical sites according to twelve distinct developmental stages—color-coded in the legend box.

**Figure 2:**
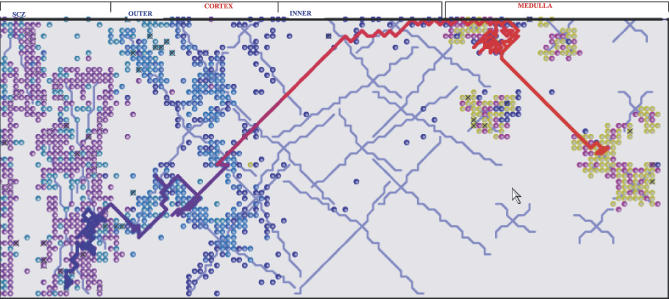
A Trace of the Path of a Single Thymocyte from Its Birth via Proliferation at the SCZ to Its Position as a Mature Cell in the Medulla We generate the visual trace by utilizing the built-in tracing tool. The trace is color-coded: the current time is highlighted in red and the beginning of the trace is enriched with blue. Intermediate times are marked as mixtures of these two colors on the trace line.

Figure 2 shows the migratory path of a single thymocyte. Both the emerging anatomy and the emerging path are faithful to experimental results [2]. Video S1 and Video S3 show dynamic versions of RA figures. These results demonstrate that the molecular data in hand suffice to generate the macroscale thymus, and that RA can reveal this cross-scalar emergence.

### Role of CXCR4 in Thymocyte Development

**Figure 3:**
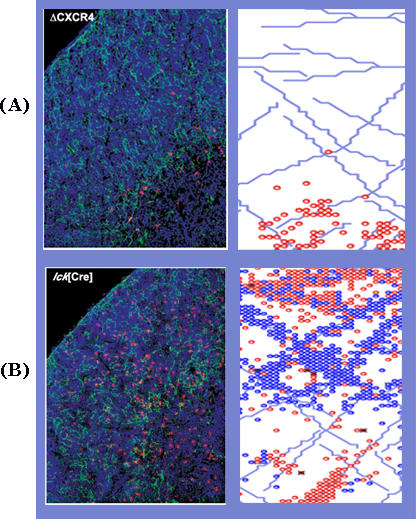
Comparing a Knockout to a Wild-Type Thymus (A) The left panel shows a figure borrowed from [19] of the effect of a CXCR4 knockout. The right panel was taken from the simulation. Red-colored cells in this right panel correspond to DN cells. The thymocytes in the experimental system, marked red fluorescence, remain very close to the CMJ, where they first entered the thymus, which is situated at the lower right corner of the plate. (B) A wild-type thymus (labeled *lck*[Cre]) shows thymocytes scattered throughout the thymic lobule both in the experimental plate from [19] on the right, and in its in silico equivalent on the left. The blue cells correspond to DP cells. DN cells and DP cells spread throughout the thymus in a normal fashion.

The effects on thymus fine anatomy of chemokine receptors CXCR4 [19] and CCR9 [20] have been studied experimentally, so we could compare an RA simulation of knocking out these receptors to the experimental results. Targeted gene deletion of CXCR4 [19] resulted in failed cortical localization and developmental arrest. Figure 3A (left) shows the thymic lobule as it was captured under the microscope [19] and as it is captured during simulation (right). In both cases, the thymocytes do not respond to CXCR4 stimulation; both Figure 3 panels show that thymocyte development gets hung up close to the cortico–medulary junction (CMJ) in the DN1 stage (labeled red). Figure 3B shows the same time frame and anatomical section in a wild-type thymus. Note that double-positive (DP) cells (blue cells in the simulation) have spread into the cortex. Unlike the static histology of the experimental model, RA provides a dynamic representation.

### Role of CCR9 in Thymocyte Development

In contrast to the CXCR4 knockout, deleting CCR9 had no major effect experimentally on intrathymic T cell development [20]. However, competitive transplantation experiments revealed that bone marrow cells from CCR9−/− mice were less efficient in repopulating the thymus of lethally irradiated Rag-1−/− mice than were bone marrow cells from littermate CCR9+/+ mice [18,20].

**Figure 4:**
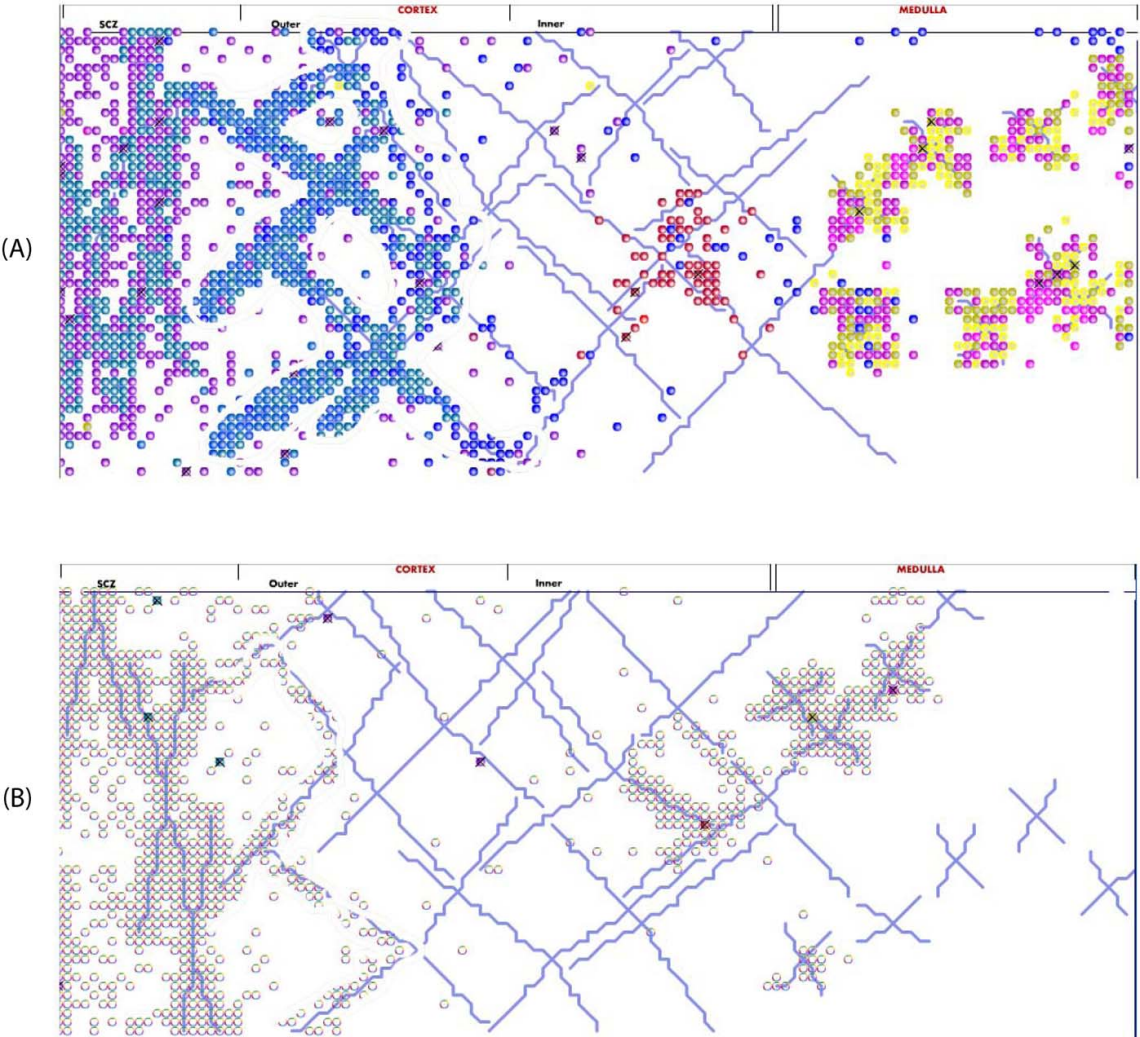
The Effect of Knocking Out CCR9 in Thymocytes (A) Shows the distribution of wild-type cells (color coded as in Figure 1). (B) Shows the altered distribution of the knockout cells (colored gray). The normal cells and the altered cells are colored differently to be compatible with Video S2, where we show the results of a competition experiment between the different cell types. The major differences are in the abilities of thymocytes to migrate from the SCZ into the cortex, after maturation from pre-DP stages. This altered behavior is responsible for the diluted numbers of thymocytes in the cortex of the knockout thymuses.

The RA simulation results, presented in Figure 4 and in Videos S1–S4, show both the influence of the lack of response to CCL25, the chemokine ligand of CCR9−/−, and the outcome of competition between CCR9−/− and wild-type cells. Figure 4A shows the normal thymus at the same time point as the altered thymus that appears in Figure 4B; the abnormal cells are coded gray here. Figure 4 is animated in Video S3, where the upper panel shows the wild-type phenotype and the lower panel shows the CCR9−/− phenotype: the CCR9−/− cells congregate around the subcortical zone (SCZ). Thymocytes at the transition from DN to DP would normally migrate towards the chemokine CCL25 and enter the cortex, but the CCR9−/− cells cannot respond to this chemokine and are blocked. The blocked thymocytes, however, can still move randomly, and population pressure pushes them away from the SCZ, so that some of them reach their next developmental checkpoint—the cortical epithelial cells—passively. These fortunate cells can then mature into their next developmental stage and migrate towards the medulla (via a different chemokine), where they can further mature (depending on further selection events) into fully functional single-positive (SP) cells. RA discloses these dynamics, surmised from static experimental histology alone.

### Interthymocyte Competition Influenced by CCR9

**Figure 5:**
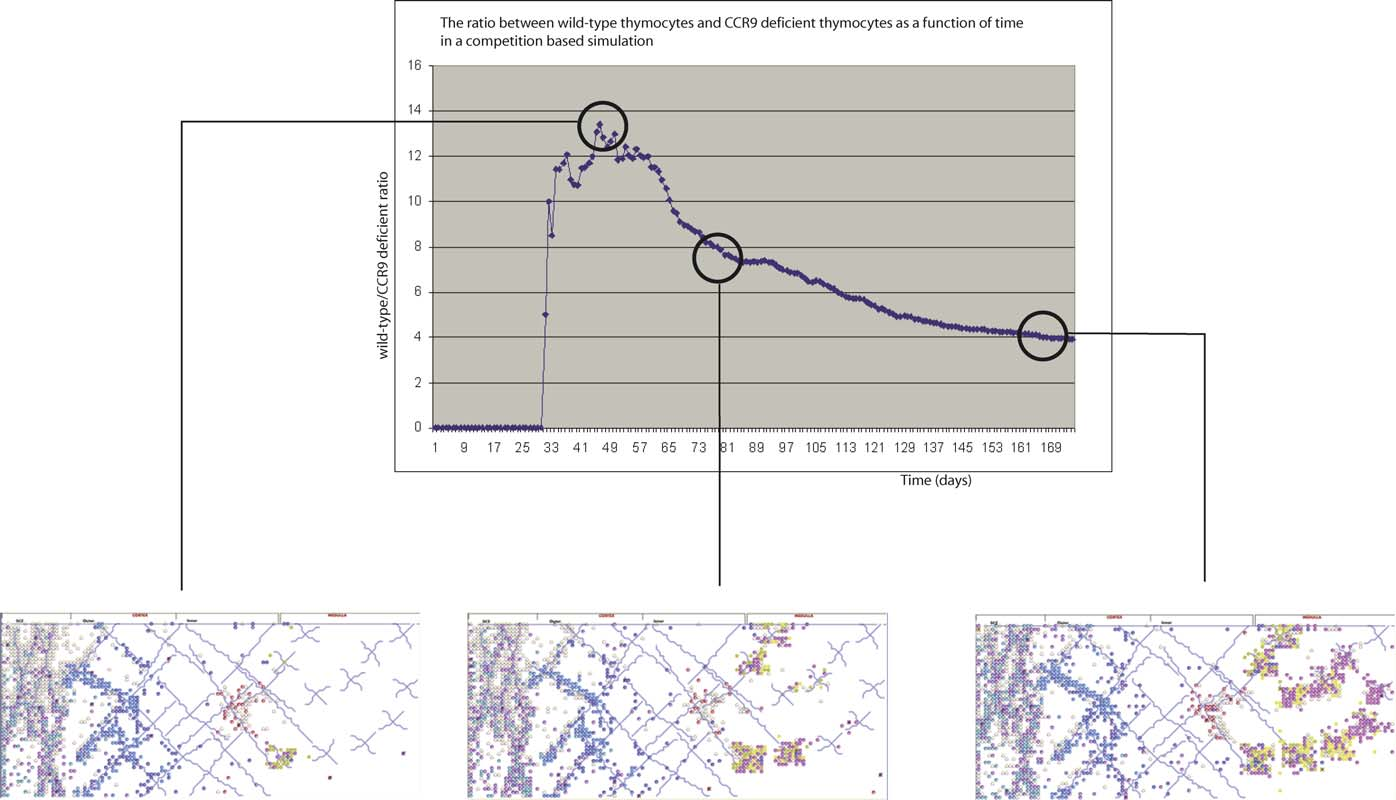
Competition In Silico between CCR9−/− Cells and Wild-Type Cells The three panels at the bottom, with context lines leading to the time-graph, show the ratios between the two cell populations developing over time. An initial peak of maturing wild-type cells is followed by a decrease and an eventual asymptotic ratio, as the buildup of random pressure of CCR9−/− cells eventually generates homeostasis. An asymptotic value of four wild-type thymocytes to every CCR9−/− thymocyte is reached. See text for further discussion.

RA also made it possible to observe the dynamics in silico of a competitive experiment, in which equal numbers of CCR9−/− and wild-type cells are seeded into the thymus: Video S2 shows that the wild-type cells survive and mature in much higher numbers. RA makes it possible to quantify the ratios between mature wild-type cells and mature CCR9−/− cells (Figure 5): we can see an initial peak of maturing wild-type cells, followed by a decrease and an eventual asymptotic ratio, as the buildup of random pressure of CCR9−/− cells eventually generates homeostasis. This competition has not yet been performed experimentally, but RA simulation predicts the outcome shown in Figure 5. Figure 5 shows an asymptotic value of four wild-type thymocytes to every CCR9−/− thymocyte. The dynamics of the asymptote and the final value are our predictions if such an experiment was to be performed. A critical point evident from Figure 5 is the overwhelming advantage that wild-type cells have immediately after seeding the thymus. Such a marked effect should be easy to witness experimentally.

### Thymocyte Competition Influences Cell Apoptosis and Velocity

**Figure 6:**
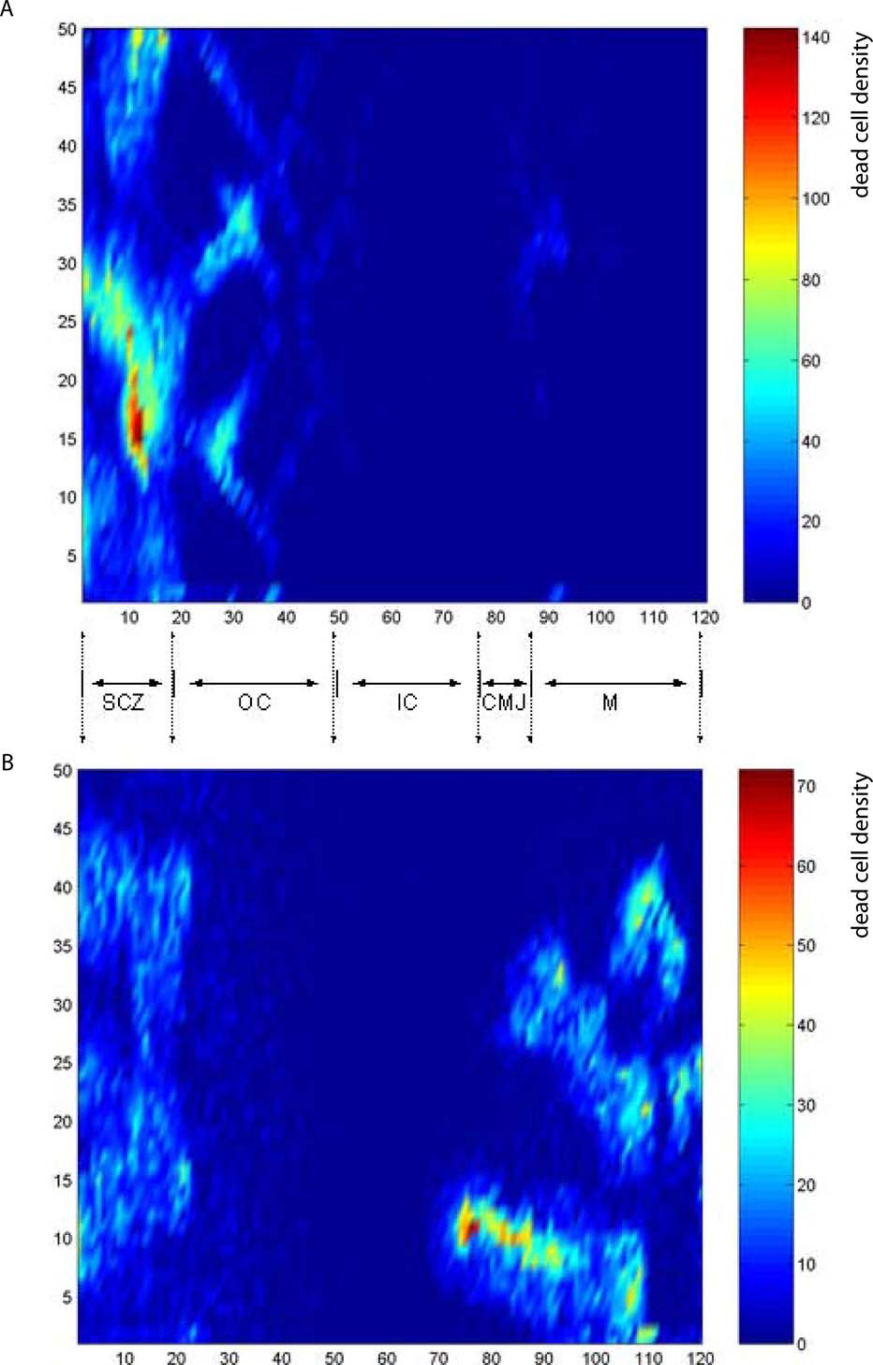
The Influence of Competition on the Pattern of Apoptosis in the Thymus We visualize the different levels of apoptosis by using different colors to show the relative numbers of apoptosing cells across the thymic grid. Red zones correspond to higher levels of apoptosis. (A) Shows the normal distribution of apoptosis primarily to the SCZ; some apoptosis is seen in the outer cortex (OC) and some in the medulla (M); the inner cortex (IC) and CMJ show relatively fewer apoptotic cells. (B) Shows the influence of removing competition between thymocytes for developmental niches. The lack of competition moves the bulk of apoptosis from the SCZ to the CMJ and the M zones. In the wild-type thymus, most of the cells die in their DP stages, in the cortex, and in the SCZ. This is in agreement with experimental results, where only 10% of cells survive to the SP stage (see review; [30]). In contrast, in the altered thymus displayed in (B), most of the cells survive to the SP stage and die in the medulla of negative selection.

Thymocytes need to traverse developmental niches; thus, when the number of thymocytes exceeds the space available for antigen presentation sites on epithelial cells, the thymocytes pile up and those waiting their turn for stimulation may undergo apoptosis from the lack of interaction [21,22]. RA makes it possible to study the function of competition by modifying interaction time constants, as shown in Video S3. The results indicate that competition is essential to normal thymic development. Figure 6A shows the normal pattern of apoptosis that occurs in the cortex in the competition-enabled thymus. Figure 6B, in contrast, shows that an abnormal pattern of apoptosis develops in a thymus free of cell competition; here, most thymocytes die of negative selection in the medulla, rather than in the cortex. The RA simulation suggests that the waiting times for interactions with cortical epithelial cells constitute a bottleneck that is a factor in normal thymus development.

**Figure 7:**
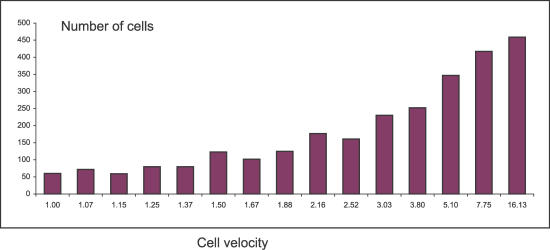
Histogram of the Different Migration Velocities of Thymocytes The faster cells are more likely to survive thymic selection by contacting the process of an epithelial cell; but slower cells also can have an advantage (see text).

RA in silico experimentation suggests that competition also selects for differential speeds of trafficking in response to chemokine gradients. Figure 7 shows that faster thymocytes enjoy greater chances of survival, at least up to a point. Nevertheless, some thymocytes that are relatively slower may avoid the negative selection suffered by some of their more speedy brothers. Thus, competition selects for a range of cell velocities, and not only for a uniformly high velocity. How selection for a range of T cell velocities might enhance defense against invaders [23] as well as for body maintenance [24] needs to be investigated.

### Competition for Niche Determines Lineage Commitment

Another prediction emerging from cell competition relates to lineage commitment. A developing thymocyte must choose whether to become an SP CD4 T cell (helper) or an SP CD8 T cell (cytotoxic). The decision-making process is obscure because mature SP CD4 and CD8 T cells evolve from precursors that are DP for both CD4 and CD8, yet CD4 cells predominate at a 2:1 ratio. Current theories of lineage commitment deal with the molecular details of the choice. The two most significant themes in the theories distinguish between an “instruction” approach and a “stochastic” approach [25]. The “instruction” approach proposes that the productive interaction of a T cell receptor with a particular major histocompatability complex (MHC) molecule, class I (for CD8) or class II (for CD4) as the case may be, rewards the thymocyte and induces a genetic choice to differentiate to the CD8 or CD4 phenotype. The more fitting T cell receptor–MHC interaction instructs the T cell. The “stochastic” approach proposes that SP CD4 or CD8 thymocytes are “randomly” generated, and are later selected according to their functional performance with the MHC. These theories attempt to found lineage choice on its molecular components aimed at showing where exactly, during development, the cell chooses its lineage [25–27].

**Figure 8:**
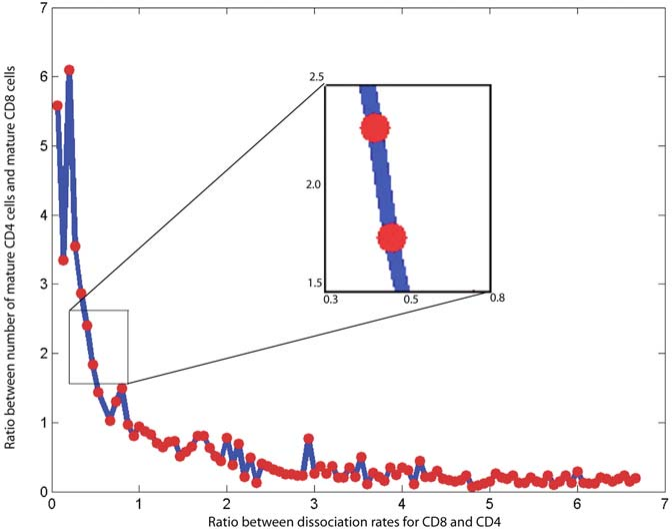
Influence of CD4 and CD8 Dissociation Rates on Lineage Commitment Ratio Measuring the ratio of CD4 mature cells to CD8 mature cells, in silico, as a function of the dissociation rate, we find that to achieve the experimentally measured 2:1 ratio, the dissociation rate of CD8 cells should be anywhere between 0.38 to 0.45 that of CD4 thymocytes. The expanded insert zooms in on this zone, which produces the experimentally observed ratio of two CD4 cells for every CD8 cell. Figure 8 shows that an increase in the dissociation rate of CD8 over CD4 could result in a CD4:CD8 population equilibrium, or even to overpopulation of the thymus with CD8 cells, depending on the relative dissociation rate.

However, the emergence of competition between thymocytes for interaction space provides a novel solution to the CD4:CD8 2:1 paradox. If the dissociation rates of CD8 cells from epithelial cells are lower than those of CD4 cells, then the CD8 cells will remain longer at their epithelial-cell interaction stations (peptide-MHC I sites). As long as a CD8 thymocyte lingers at a peptide-MHC 1 niche, this niche is unavailable for other, competing CD8 thymocytes. CD8 thymocytes, we propose, do not compete with the CD4 thymocytes, because CD4 thymocytes compete among themselves for stimulation by interacting with peptide–MHC II stations on epithelial cells. We tested the outcome on lineage frequency of simulating different dissociation rates for interactions between epithelial cells and CD4 and CD8 thymocytes. The results are shown in Figure 8. It can be seen that about two-thirds of thymocytes will mature into CD4 T cells and one-third into CD8 T cells (the de facto ratio) when the dissociation rate of CD8 thymocytes is 1.7 to 3.3 times slower than the dissociation rate of CD4 thymocytes. A relatively greater avidity of CD8 cells for epithelial cell niches (by 1.7–3.3) would generate the observed lineage predominance of CD4 T cells.

## Discussion

RA analysis of thymocyte development sheds new light on the dynamic relationship between molecules and cells in generating the structure and function of the thymus organ. First, we can see that the existing body of data, however discrete and piecemeal, can be integrated by RA simulation into a representation of the functional anatomy of the thymus seen in histologic sections. What we know about cells and molecules can indeed account for what we see; the macroscale organ emerges from the microscale mass of data in hand. In this regard, RA can be said to validate the database. Note, however, that classical histologic sections are two-dimensional slices of a three-dimensional organ frozen in time; RA simulation adds the dimension of time—dynamics—and so shows us the formative power of the dynamic flux of cells, molecules, and interactions that give rise to the higher-scale organ. In another project involving a different organ, we are currently extending RA simulations to accommodate the third dimension in space; hopefully, the added complexity of the representation will enhance our understanding of the biology.

Second, RA simulation offers novel explanations for the observed outcomes of experimental intervention. In our case, for example, RA simulation suggested that the lack of phenotype observed in mice with CCR9 knocked out (CCR9−/−) might be explained by dynamic compensation through population pressure. RA simulation also explains the competitive growth advantage enjoyed by wild-type cells over CCR9−/− cells. Indeed, overexpression of CCR9 on thymocytes leads to an in vivo phenotype that can be explained by RA as an untimely attraction of the thymocytes by cortical epithelial cells. RA simulation also suggests that the absence of thymic output resulting from CXCR4 inhibition can be attributed to the nonmigratory behavior of cells entering the thymus.

Third, the visualization of cell dynamics through RA provides a view of emergent physiology. Although the thymus is packed full of cells, the existence of competition among thymocytes for space and stimulation has not been a subject for experimentation or even discussion; competition is simply not seen in static histologic sections. Since competition was not recorded in the database, we did not explicitly program competition into our model. Nevertheless, cell competition emerged before our very eyes as we witnessed, via RA, the animated struggle between individual thymocytes for productive interactions with thymic epithelial cells. In silico manipulation of various parameters suggested that thymocyte competition might function as an important factor in three emergent properties of T cell maturation: the functional anatomy of the thymus, the selection of thymocytes with a range of migratory velocities, and the relative preponderance of SP CD4 T cells. Obviously, these suggestions require experimental validation*.* Irrespective of the outcome, however, the animation arm of RA, in providing a higher-scale view of complex emergent properties [28], can alert us to new questions for experimentation. Ultimately, we would like to model a complete biological system—an entire cell, organ, or organism—in a way that is sufficiently realistic so as to be able to test the role of any known fact about the system. This goal has been formulated as the ability of a model to pass a sort of Turing test, and can be viewed as taking to the utmost limit the notion of prediction, confirmation, and verification of emergent properties; see [29].

## Materials and Methods

### Simulation.

The RA simulation was written in C++ using the Rhapsody tool, and so RA code was generated by Rhapsody's code-generation engine, initiated by the language of Statecharts. To this automatically generated code, manually encoded objects and function were added. RA is the bridge made between the running simulation and the animation. Communication is made over a TCP/IP connection between a server implementing the Statecharts simulation and built-in animation functions in Flash. We used Matlab to analyze populations and population-level behavior. See [6] for further details.

## Supporting Information

Text S1 Collection of References Used in the Thymus Simulation (74 KB PDF)

Video S1 Survival of Wild-Type Cells in Comparison with Mutated Cells (57.8 MB AVI)

Video S2 Migration Patterns in Wild-Type and Mutated Cells (10.9 MB MOV)

Video S3 Homeostasis in the Wild-Type Thymus (6.3 MB MOV)

Video S4 Following the Migratory Trail of a Single Thymocyte (3.3 MB AVI)

## Abbreviations

CMJ: cortico–medulary junction
DN: double-negative
DP: double-positive
MHC: major histocompatibility complex
RA: reactive animation
SCZ: subcortical zone
SP: single-positive

## References

[pcbi-0030013-b001] Anderson G, Jenkinson EJ (2001). Lymphostromal interactions in thymic development and function. Nat Rev Immunol.

[pcbi-0030013-b002] Petrie HT (2003). Cell migration and the control of post-natal T-cell lymphopoiesis in the thymus. Nat Rev Immunol.

[pcbi-0030013-b003] Janeway C (2001). Immunobiology: The immune system in health and disease.

[pcbi-0030013-b004] Takahama Y (2006). Journey through the thymus: Stromal guides for T-cell development and selection. Nat Rev Immunol.

[pcbi-0030013-b005] Efroni S, Harel D, Cohen IR (2003). Toward rigorous comprehension of biological complexity: Modeling, execution, and visualization of thymic T-cell maturation. Genome Res.

[pcbi-0030013-b006] Efroni S, Harel D, Cohen IR (2005). Reactive animation: Realistic modeling of complex dynamic systems. Computer.

[pcbi-0030013-b007] Harel D, Efroni S, Cohen IR (2003). Reactive animation. Lect Notes Comput Sci.

[pcbi-0030013-b008] Harel D (1987). Statecharts: A visual formalism for complex systems. Sci Comput Programming.

[pcbi-0030013-b009] Sulzer B, Perelson AS (1996). Equilibrium binding of multivalent ligands to cells: Effects of cell and receptor density. Math Biosci.

[pcbi-0030013-b010] De Boer RJ, Perelson AS (1994). T cell repertoires and competitive exclusion. J Theor Biol.

[pcbi-0030013-b011] Kohler B, Puzone R, Seiden PE, Celada F (2000). A systematic approach to vaccine complexity using an automaton model of the cellular and humoral immune system. I. Viral characteristics and polarized responses. Vaccine.

[pcbi-0030013-b012] Li QJ, Dinner AR, Qi S, Irvine DJ, Huppa JB (2004). CD4 enhances T cell sensitivity to antigen by coordinating Lck accumulation at the immunological synapse. Nat Immunol.

[pcbi-0030013-b013] Chakraborty AK (2002). How and why does the immunological synapse form? Physical chemistry meets cell biology. Sci STKE.

[pcbi-0030013-b014] Schatz DG (2004). Antigen receptor genes and the evolution of a recombinase. Semin Immunol.

[pcbi-0030013-b015] Meier-Schellersheim M (1999). SIMMUNE, a tool for simulating and analyzing immune system behavior.

[pcbi-0030013-b016] Meier-Schellersheim M, Xu X, Angermann B, Kunkel EJ, Jin T (2006). Key role of local regulation in chemosensing revealed by a new molecular interaction–based modeling method. PLoS Comput Biol.

[pcbi-0030013-b017] Rothenberg EV, Taghon T (2005). Molecular genetics of T cell development. Annu Rev Immunol.

[pcbi-0030013-b018] Wurbel MA, Philippe JM, Nguyen C, Victorero G, Freeman T (2000). The chemokine TECK is expressed by thymic and intestinal epithelial cells and attracts double- and single-positive thymocytes expressing the TECK receptor CCR9. Eur J Immunol.

[pcbi-0030013-b019] Plotkin J, Prockop SE, Lepique A, Petrie HT (2003). Critical role for CXCR4 signaling in progenitor localization and T cell differentiation in the postnatal thymus. J Immunol.

[pcbi-0030013-b020] Uehara S, Grinberg A, Farber JM, Love PE (2002). A role for CCR9 in T lymphocyte development and migration. J Immunol.

[pcbi-0030013-b021] Ashwell JD, Lu FW, Vacchio MS (2000). Glucocorticoids in T cell development and function*. Annu Rev Immunol.

[pcbi-0030013-b022] Lu FW, Yasutomo K, Goodman GB, McHeyzer-Williams LJ, McHeyzer-Williams MG (2000). Thymocyte resistance to glucocorticoids leads to antigen-specific unresponsiveness due to “holes” in the T cell repertoire. Immunity.

[pcbi-0030013-b023] Singer AL, Koretzky GA (2002). Control of T cell function by positive and negative regulators. Science.

[pcbi-0030013-b024] Cohen IR (2000). Discrimination and dialogue in the immune system. Semin Immunol.

[pcbi-0030013-b025] Germain RN (2002). T-cell development and the CD4–CD8 lineage decision. Nat Rev Immunol.

[pcbi-0030013-b026] He X, He X, Dave VP, Zhang Y, Hua X (2005). The zinc finger transcription factor Th-POK regulates CD4 versus CD8 T-cell lineage commitment. Nature.

[pcbi-0030013-b027] Bosselut R, Guinter TI, Sharrow SO, Singer A (2003). Unraveling a revealing paradox: Why major histocompatibility complex I-signaled thymocytes “paradoxically” appear as CD4+8lo transitional cells during positive selection of CD8+ T cells. J Exp Med.

[pcbi-0030013-b028] Cohen IR (2000). Tending Adam's garden: Evolving the cognitive immune self.

[pcbi-0030013-b029] Harel D (2005). A Turing-like test for biological modeling. Nat Biotechnol.

[pcbi-0030013-b030] Chen W (2004). The late stage of T cell development within mouse thymus. Cell Mol Immunol.

