# Supplementary material for: Emergent Dynamics of Thymocyte Development and Lineage Determination
Source: PLoS Comput Biol. 2007 Jan 26;3(1):e13. doi: 10.1371/journal.pcbi.0030013 (PMC1782042; doi:10.1371/journal.pcbi.0030013)
Supplement: Text S1 — (74 KB PDF) [file pcbi.0030013.se001.pdf]

Adhesion to epithelial cells
(Dalmau, Freitas et al. 1999)

Apoptosis in the thymus
- (Imachi, Murao et al. 2000)
- (van Lent, Licht et al. 2001)
- (Hu, Punturieri et al. 2002)
- (Samms, Martinez et al. 2001)
- (Platt, Suzuki et al. 2000)
- (Terpstra, Kondratenko et al. 1997)
- (Platt, Suzuki et al. 1996)
- (Sohn, Rajpal et al. 2003)
- (Nauta, Daha et al. 2003)

CD4 CD8 choice
- (Ellmeier, Sawada et al. 1999)
- (Bosselut, Guinter et al. 2003)
- (Lovatt, Yang et al. 2000)
- (Correia-Neves, Mathis et al. 2001)
- (Hutchinson, Wooldridge et al. 2003)
- (Germain 2002)
- (Fink 2003)
- (Canelles, Park et al. 2003)
- (Bosselut, Feigenbaum et al. 2001)

Chemokines - general
- (Lucas and Greaves 2001)
- (Kunkel and Butcher 2002)
- (Mora, Bono et al. 2003)
- (Gao and Metz 2003)
- (Fernandez and Lolis 2002)
- (Murphy, Baggiolini et al. 2000)
- (Rossi and Zlotnik 2000)

Chemokine – thymus
- (Uehara, Song et al. 2002)
- (Annunziato, Romagnani et al. 2000)
- (Feng, Woodside et al. 2002)
- (Hernandez-Lopez, Varas et al. 2002)
- (Romagnani, Annunziato et al. 2001)
- (Suzuki, Nakata et al. 1998)
- (Poznansky, Olszak et al. 2002)
- (Zaitseva, Kawamura et al. 2002)
- (Wurbel, Malissen et al. 2001)
- (Ara, Itoi et al. 2003)
- (Suzuki, Sawa et al. 1999)
- (Aiuti, Tavian et al. 1999)
- (Adachi, Kuwata et al. 2001)
- (Kim and Broxmeyer 1999)
- (Savino, Mendes-da-Cruz et al. 2002)
- (Jin, Fu et al. 2003)
- (Wurbel, Philippe et al. 2000)

Complex systems
- (Carlson and Doyle 2002)
- (Newman 2000)

Cytokines
- (Yu, Erman et al. 2003)
- (Di Santo and Rodewald 1998)
- (Kawamoto, Ohmura et al. 2003)

Dendritic cells
- (Brocker 1999)
- (Duncan, Capetanakis et al. 2002)
- (Donskoy and Goldschneider 2003)
- (Delaney, Sykulev et al. 1998)
- (Zinkernagel and Althage 1999)
- (Mellman and Steinman 2001)
- (Liu 2001)
- (Keller 2001)
- (Lipscomb and Masten 2002)

Fibroblasts
- (Lilic, Santori et al. 2002)

Reviews
- (Starr, Jameson et al. 2003)
- (Fabbri, Smart et al. 2003)
- (Bendelac, Bonneville et al. 2001)
- (Lehar and Bevan 2002)
- (Savino, Ayres Martins et al. 2003)
- (Robey and Schlissel 2003)
- (von Boehmer, Aifantis et al. 2003)
- (Mak, Penninger et al. 2001)
- (Anderton and Wraith 2002)
- (Anderson and Jenkinson 2001)
- (Spits 2002)
- (Love and Chan 2003)
- (Sercarz and Maverakis 2003)

IL7
- (Dus, Krawczenko et al. 2003)

Immunological repertoire
- (Livak and Petrie 2001)
- (Bergmann, Tong et al. 1994)
- (Kreuwel and Sherman 2001)

Integrins
- (Schmeissner, Xie et al. 2001)
- (Banwell, Partington et al. 2000)

Knock outs
- (Wurbel, Malissen et al. 2001)
- (Legrand and Freitas 2001)
- (Taylor, Kimbrell et al. 2001)
- (Pitkanen and Peterson 2003)
- (Lee, Ramos et al. 2003)
- (Blank, Brown et al. 2003)
- (Souto, Brito et al. 2003)
- (Christianson, Greiner et al. 2002)
- (Fraser, Ferguson et al. 2002)

Medulla
- (Farr, Dooley et al. 2002)

Immunological Methods
- (Arion, Moskvina et al. 2001)
- (Gordon, Duckett et al. 2003)

MHCs
- (Arudchelvan, Nishimura et al. 2002)
- (Douek and Altmann 2000)
- (Arudchelvan, Tokuda et al. 2002)
- (Arudchelvan, Nishimura et al. 2003)
- (Vukmanovic, Neubert et al. 2003)
- (Kangueane, Sakharkar et al. 2001)

Microarray
- (Ramialison, Mohr et al. 2002)

Modeling
- (Malissen 2003)
- (Lee, Dinner et al. 2003)
- (de Jong 2002)
- (Deenick, Gett et al. 2003)
- (Gett and Hodgkin 2000)
- (Chakraborty, Dustin et al. 2003)

Pre stages
- (Vanhecke, Verhasselt et al. 1997)

Negative Selection
- (Cho, Edmondson et al. 2003)
- (Teshima, Reddy et al. 2003)
- (Ohashi 2003)
- (Palmer 2003)
- (Sprent and Kishimoto 2002)

Notch
- (Harman, Jenkinson et al. 2003)
- (Bellavia, Campese et al. 2003)
- (Harman, Jenkinson et al. 2003)
- (Artavanis-Tsakonas, Rand et al. 1999)

Petrie
- (Baldwin, Deutsch et al. 1993)
- (Petrie, Livak et al. 1993)
- (Petrie, Strasser et al. 1993)
- (Dudley, Petrie et al. 1994)
- (Wayne, Suh et al. 1994)
- (Livak, Petrie et al. 1995)
- (Petrie, Livak et al. 1995)
- (Burtrum, Kim et al. 1996)
- (Mazel, Burtrum et al. 1996)
- (Tourigny, Mazel et al. 1997)
- (Murgia, Blaikie et al. 1998)
- (King, Tolosa et al. 1999)
- (Lind, Wayne et al. 1999)
- (Littman, Sun et al. 1999)
- (Livak, Tourigny et al. 1999)
- (Brady, Lee et al. 2000)
- (Brungart, Holmberg et al. 2000)
- (Livak, Burtrum et al. 2000)
- (Petrie 2000
- (Petrie, Tourigny et al. 2000)
- (Petrie-Hanson and Ainsworth 2000)
- (Prockop and Petrie 2000)
- (Lind, Prockop et al. 2001)
- (Livak and Petrie 2001)
- (Petrie-Hanson and Ainsworth 2001)
- (Yannoutsos, Wilson et al. 2001)
- (Livak and Petrie 2002)
- (Petrie 2002)
- (Petrie and Van Ewijk 2002)
- (Prockop, Palencia et al. 2002)
- (Gordon, Duckett et al. 2003)
- (Lepique, Palencia et al. 2003)
- (Plotkin, Prockop et al. 2003)
- (Porritt, Gordon et al. 2003)

Positive Selection
- (van der Merwe and Davis 2003)
- (Viret, He et al. 2003)
- (Starr, Jameson et al. 2003)
- (Correia-Neves, Mathis et al. 2001)
- (Holmberg, Mariathasan et al. 2003)
- (Janeway 1999)
- (Anderson, Hare et al. 1999)
- (Wilkinson, Anderson et al. 1995)
- (Alberola-Ila and Hernandez-Hoyos 2003)

Pre TCR
- (Ito, Arai et al. 2002)
- (Carrasco, Navarro et al. 2002)(Michie and Zuniga-Pflucker 2002)

Specificity
- (von Greyerz, Bultemann et al. 2001)
- (Zhao, Gran et al. 2001)
- (Wilson, Pinilla et al. 1999)
- (Holler and Kranz 2003)
- (Jacobsen, Cepok et al. 2001)
- (Borras, Martin et al. 2002)

Regulation
- (Powrie and Maloy 2003)
- (McHugh and Shevach 2002)
- (Stephens and Ignatowicz 2003)
- (Wang, Wang-Zhu et al. 2002)
- (Seddon and Mason 2000)
- (Apostolou, Sarukhan et al. 2002)
- (Shevach 2002)
- (Schwartz and Kipnis 2002)

T – T interactions
- (Choi, Park et al. 1997)
- (Choi, Bae et al. 2001)

Advanced technique thymic Microscopy
- (Miller, Wei et al. 2002)
- (Stoll, Delon et al. 2002)
- (Bousso, Bhakta et al. 2002)
- (Irvine, Purbhoo et al. 2002)
- (Delon, Stoll et al. 2002)

TCR binding
- (van der Merwe and Davis 2003)
- (Lyons, Lieberman et al. 1996)
- (Wang and Eck 2003)

TCR complex
- (Call, Pyrdol et al. 2002)

Thymic development – general
- (Hare, Wilkinson et al. 1998)
- (Anderson, Harman et al. 2000)
- (Norment and Bevan 2000)
- (Penit, Lucas et al. 1995)
- (Tourigny, Mazel et al. 1997)
- (Clevers 2003)
- (Hunig, Torres-Nagel et al. 2001)
- (Sebzda, Mariathasan et al. 1999)

Epithelial cells
- (Bennett, Farley et al. 2002)

Antigen expression
- (Hanahan 1998)

Migration
- (Uehara, Song et al. 2002)
- (Kremer, Carramolino et al. 2001)
- (Annunziato, Romagnani et al. 2000)
- (Wurbel, Philippe et al. 2000)
- (Luster 1998)
- (Hernandez-Lopez, Varas et al. 2002)
- (Romagnani, Annunziato et al. 2001)
- (Norment, Bogatzki et al. 2000)
- (Carramolino, Zaballos et al. 2001)
- (Cyster 2002)
- (von Andrian and Mackay 2000)
- (Zabel, Agace et al. 1999)
- (Chantry, Romagnani et al. 1999)
- (Youn, Kim et al. 1999)
- (Zaitseva, Kawamura et al. 2002)
- (Uehara, Grinberg et al. 2002)
- (Zaitseva, Lee et al. 1998)
- (Bleul and Boehm 2000)
- (Kim, Pelus et al. 1998)
- (Zaballos, Gutierrez et al. 1999)
- (Suzuki, Sawa et al. 1999)
- (Taylor, Kimbrell et al. 2001)
- (Norment and Bevan 2000)
- (Youn, Kim et al. 1999)
- (Campbell, Pan et al. 1999)
- (Annunziato, Romagnani et al. 2001)
- (Zlotnik and Yoshie 2000)
- (Kunkel and Butcher 2002)
- (Ueno, Hara et al. 2002)
- (Savino, Mendes-da-Cruz et al. 2002)
- (Scollay and Godfrey 1995)

Thymic nurse cell
- (Pezzano, Samms et al. 2001)
- (Oliveira-dos-Santos, Rieker-Geley et al. 1997)
- (Samms, Philp et al. 1999)
- (Brelinska and Warchol 1997)

Thymic Selection
- (Zippelius, Pittet et al. 2002)
- (Delaney, Sykulev et al. 1998)
- (Mariathasan, Zakarian et al. 2001)
- (Brocker, Riedinger et al. 1997)

Thymic stroma expression
- (Oosterwegel, Haks et al. 1997)
- (Klug, Carter et al. 1998)
- (Felli, Maroder et al. 1999)

Thymus Microscopy
- (Arudchelvan, Tokuda et al. 2002)
- (De Waal and Rademakers 1997)
- (Schuurman, Kuper et al. 1997)
- (Nabarra, Mulotte et al. 2001)
- (Von Gaudecker, Kendall et al. 1997)

Cell Cycle
- (Lin and Desiderio 1994)

Adachi, S., T. Kuwata, et al. (2001). "Induction of CCR7 expression in thymocytes requires both ERK signal and Ca(2+) signal." Biochem Biophys Res Commun **288**(5): 1188-93.

Aiuti, A., M. Tavian, et al. (1999). "Expression of CXCR4, the receptor for stromal cell-derived factor-1 on fetal and adult human lympho-hematopoietic progenitors." Eur J Immunol **29**(6): 1823-31.

Alberola-Ila, J. and G. Hernandez-Hoyos (2003). "The Ras/MAPK cascade and the control of positive selection." Immunol Rev **191**: 79-96.

Anderson, G., K. J. Hare, et al. (1999). "Positive selection of thymocytes: the long and winding road." Immunol Today **20**(10): 463-8.

Anderson, G., B. C. Harman, et al. (2000). "Microenvironmental regulation of T cell development in the thymus." Semin Immunol **12**(5): 457-64.

Anderson, G. and E. J. Jenkinson (2001). "Lymphostromal interactions in thymic development and function." Nat Rev Immunol **1**(1): 31-40.

Anderton, S. M. and D. C. Wraith (2002). "Selection and fine-tuning of the autoimmune T-cell repertoire." Nat Rev Immunol **2**(7): 487-98.

Annunziato, F., P. Romagnani, et al. (2000). "Macrophage-derived chemokine and EBI1-ligand chemokine attract human thymocytes in different stage of development and are produced by distinct subsets of medullary epithelial cells: possible implications for negative selection." J Immunol **165**(1): 238-46.

Annunziato, F., P. Romagnani, et al. (2001). "Chemokines and lymphopoiesis in human thymus." Trends Immunol **22**(5): 277-81.

Apostolou, I., A. Sarukhan, et al. (2002). "Origin of regulatory T cells with known specificity for antigen." Nat Immunol **3**(8): 756-63.

Ara, T., M. Itoi, et al. (2003). "A role of CXC chemokine ligand 12/stromal cell-derived factor-1/pre-B cell growth stimulating factor and its receptor CXCR4 in fetal and adult T cell development in vivo." J Immunol **170**(9): 4649-55.

Arion, V. Y., S. N. Moskvina, et al. (2001). "Evaluation of Thymus Functional State by Non-Invasive Method." Russ J Immunol **6**(1): 77-80.

Artavanis-Tsakonas, S., M. D. Rand, et al. (1999). "Notch signaling: cell fate control and signal integration in development." Science **284**(5415): 770-6.

Arudchelvan, Y., Y. Nishimura, et al. (2002). "Differential expression of MHC class II antigens and cathepsin L by subtypes of cortical epithelial cells in the rat thymus: an immunoelectron microscopic study." J Electron Microsc (Tokyo) **51**(3): 173-81.

Arudchelvan, Y., Y. Nishimura, et al. (2003). "Identification and characterization of major histocompatibility complex class II compartments in cortical thymic epithelial cells." Anat Rec **274A**(1): 798-806.

Arudchelvan, Y., N. Tokuda, et al. (2002). "Spatial relation between major histocompatibility complex-restricted antigen receptor-bearing thymocytes and subtypes of thymic epithelial cells." Anat Rec **267**(2): 131-6.

Baldwin, J. T., S. Deutsch, et al. (1993). "Determination of principal reynolds stresses in pulsatile flows after elliptical filtering of discrete velocity measurements." J Biomech Eng **115**(4A): 396-403.

Banwell, C. M., K. M. Partington, et al. (2000). "Studies on the role of IL-7 presentation by mesenchymal fibroblasts during early thymocyte development." Eur J Immunol **30**(8): 2125-9.

Bellavia, D., A. F. Campese, et al. (2003). "Notch3, another Notch in T cell development." Semin Immunol **15**(2): 107-12.

Bendelac, A., M. Bonneville, et al. (2001). "Autoreactivity by design: innate B and T lymphocytes." Nat Rev Immunol **1**(3): 177-86.
Bennett, A. R., A. Farley, et al. (2002). "Identification and characterization of thymic epithelial progenitor cells." Immunity **16**(6): 803-14.
Bergmann, C. C., L. Tong, et al. (1994). "Cytotoxic T cell repertoire selection. A single amino acid determines alternative class I restriction." J Immunol **152**(12): 5603-12.
Blank, C., I. Brown, et al. (2003). "Absence of programmed death receptor 1 alters thymic development and enhances generation of CD4/CD8 double-negative TCR-transgenic T cells." J Immunol **171**(9): 4574-81.
Bleul, C. C. and T. Boehm (2000). "Chemokines define distinct microenvironments in the developing thymus." Eur J Immunol **30**(12): 3371-9.
Borras, E., R. Martin, et al. (2002). "Findings on T cell specificity revealed by synthetic combinatorial libraries." J Immunol Methods **267**(1): 79-97.
Bosselut, R., L. Feigenbaum, et al. (2001). "Strength of signaling by CD4 and CD8 coreceptor tails determines the number but not the lineage direction of positively selected thymocytes." Immunity **14**(4): 483-94.
Bosselut, R., T. I. Guinter, et al. (2003). "Unraveling a revealing paradox: Why major histocompatibility complex I-signaled thymocytes "paradoxically" appear as CD4+8lo transitional cells during positive selection of CD8+ T cells." J Exp Med **197**(12): 1709-19.
Bousso, P., N. R. Bhakta, et al. (2002). "Dynamics of thymocyte-stromal cell interactions visualized by two-photon microscopy." Science **296**(5574): 1876-80.
Brady, M. S., F. Lee, et al. (2000). "CD4(+) T cells kill HLA-class-II-antigen-positive melanoma cells presenting peptide in vitro." Cancer Immunol Immunother **48**(11): 621-6.
Brelinska, R. and J. B. Warchol (1997). "Thymic nurse cells: their functional ultrastructure." Microsc Res Tech **38**(3): 250-66.
Brocker, T. (1999). "The role of dendritic cells in T cell selection and survival." J Leukoc Biol **66**(2): 331-5.
Brocker, T., M. Riedinger, et al. (1997). "Targeted expression of major histocompatibility complex (MHC) class II molecules demonstrates that dendritic cells can induce negative but not positive selection of thymocytes in vivo." J Exp Med **185**(3): 541-50.
Brungart, T. A., W. J. Holmberg, et al. (2000). "The scaling of the wall pressure fluctuations in polymer-modified turbulent boundary layer flow." J Acoust Soc Am **108**(1): 71-5.
Burtrum, D. B., S. Kim, et al. (1996). "TCR gene recombination and alpha beta-gamma delta lineage divergence: productive TCR-beta rearrangement is neither exclusive nor preclusive of gamma delta cell development." J Immunol **157**(10): 4293-6.
Call, M. E., J. Pyrdol, et al. (2002). "The organizing principle in the formation of the T cell receptor-CD3 complex." Cell **111**(7): 967-79.
Campbell, J. J., J. Pan, et al. (1999). "Cutting edge: developmental switches in chemokine responses during T cell maturation." J Immunol **163**(5): 2353-7.
Canelles, M., M. L. Park, et al. (2003). "The influence of the thymic environment on the CD4-versus-CD8 T lineage decision." Nat Immunol **4**(8): 756-64.
Carlson, J. M. and J. Doyle (2002). "Complexity and robustness." Proc Natl Acad Sci U S A **99 Suppl 1**: 2538-45.

Carramolino, L., A. Zaballos, et al. (2001). "Expression of CCR9 beta-chemokine receptor is modulated in thymocyte differentiation and is selectively maintained in CD8(+) T cells from secondary lymphoid organs." Blood **97**(4): 850-7.
Carrasco, Y. R., M. N. Navarro, et al. (2002). "Regulation of surface expression of the human pre-T cell receptor complex." Semin Immunol **14**(5): 325-34.
Chakraborty, A. K., M. L. Dustin, et al. (2003). "In silico models for cellular and molecular immunology: successes, promises and challenges." Nat Immunol **4**(10): 933-6.
Chantry, D., P. Romagnani, et al. (1999). "Macrophage-derived chemokine is localized to thymic medullary epithelial cells and is a chemoattractant for CD3(+), CD4(+), CD8(low) thymocytes." Blood **94**(6): 1890-8.
Cho, H. J., S. G. Edmondson, et al. (2003). "Cutting edge: identification of the targets of clonal deletion in an unmanipulated thymus." J Immunol **170**(1): 10-3.
Choi, E. Y., Y. Bae, et al. (2001). "T-T interaction during thymic selection." Cell Mol Biol (Noisy-le-grand) **47**(1): 135-43.
Choi, E. Y., W. S. Park, et al. (1997). "Thymocytes positively select thymocytes in human system." Hum Immunol **54**(1): 15-20.
Christianson, S. W., D. L. Greiner, et al. (2002). "T cell developmental defects in 'viable motheaten' mice deficient in SHP-1 protein-tyrosine phosphatase. Developmental defects are corrected in vitro in the presence of normal hematopoietic-origin stromal cells and in vivo by exogenous IL-7." J Autoimmun **18**(2): 119-30.
Clevers, H. (2003). "Thymocyte development: drama without Brahma." Immunity **19**(2): 157-8.
Correia-Neves, M., D. Mathis, et al. (2001). "A molecular chart of thymocyte positive selection." Eur J Immunol **31**(9): 2583-92.
Cyster, J. G. (2002). "Chemorepulsion and thymocyte emigration." J Clin Invest **109**(8): 1011-2.
Dalmau, S. R., C. S. Freitas, et al. (1999). "Upregulated expression of fibronectin receptors underlines the adhesive capability of thymocytes to thymic epithelial cells during the early stages of differentiation: lessons from sublethally irradiated mice." Blood **93**(3): 974-90.
de Jong, H. (2002). "Modeling and simulation of genetic regulatory systems: a literature review." J Comput Biol **9**(1): 67-103.
De Waal, E. J. and L. H. Rademakers (1997). "Heterogeneity of epithelial cells in the rat thymus." Microsc Res Tech **38**(3): 227-36.
Deenick, E. K., A. V. Gett, et al. (2003). "Stochastic model of T cell proliferation: a calculus revealing IL-2 regulation of precursor frequencies, cell cycle time, and survival." J Immunol **170**(10): 4963-72.
Delaney, J. R., Y. Sykulev, et al. (1998). "Differences in the level of expression of class I major histocompatibility complex proteins on thymic epithelial and dendritic cells influence the decision of immature thymocytes between positive and negative selection." Proc Natl Acad Sci U S A **95**(9): 5235-40.
Di Santo, J. P. and H. R. Rodewald (1998). "In vivo roles of receptor tyrosine kinases and cytokine receptors in early thymocyte development." Curr Opin Immunol **10**(2): 196-207.
Donskoy, E. and I. Goldschneider (2003). "Two developmentally distinct populations of dendritic cells inhabit the adult mouse thymus: demonstration by

differential importation of hematogenous precursors under steady state conditions." J Immunol **170**(7): 3514-21.
Douek, D. C. and D. M. Altmann (2000). "T-cell apoptosis and differential human leucocyte antigen class II expression in human thymus." Immunology **99**(2): 249-56.
Dudley, E. C., H. T. Petrie, et al. (1994). "T cell receptor beta chain gene rearrangement and selection during thymocyte development in adult mice." Immunity **1**(2): 83-93.
Duncan, S. R., N. G. Capetanakis, et al. (2002). "Thymic dendritic cells traffic to thymi of allogeneic recipients and prolong graft survival." J Clin Invest **109**(6): 755-64.
Dus, D., A. Krawczenko, et al. (2003). "IL-7 receptor is present on human microvascular endothelial cells." Immunol Lett **86**(2): 163-8.
Ellmeier, W., S. Sawada, et al. (1999). "The regulation of CD4 and CD8 coreceptor gene expression during T cell development." Annu Rev Immunol **17**: 523-54.
Fabbri, M., C. Smart, et al. (2003). "T lymphocytes." Int J Biochem Cell Biol **35**(7): 1004-8.
Farr, A. G., J. L. Dooley, et al. (2002). "Organization of thymic medullary epithelial heterogeneity: implications for mechanisms of epithelial differentiation." Immunol Rev **189**: 20-7.
Felli, M. P., M. Maroder, et al. (1999). "Expression pattern of notch1, 2 and 3 and Jagged1 and 2 in lymphoid and stromal thymus components: distinct ligand-receptor interactions in intrathymic T cell development." Int Immunol **11**(7): 1017-25.
Feng, C., K. J. Woodside, et al. (2002). "A potential role for CD69 in thymocyte emigration." Int Immunol **14**(6): 535-44.
Fernandez, E. J. and E. Lolis (2002). "Structure, function, and inhibition of chemokines." Annu Rev Pharmacol Toxicol **42**: 469-99.
Fink, P. J. (2003). "Deadbeat neighbors influence thymocyte lineage commitment." Nat Immunol **4**(8): 727-8.
Fraser, C., N. M. Ferguson, et al. (2002). "Antigen-driven T-cell turnover." J Theor Biol **219**(2): 177-92.
Gao, Z. and W. A. Metz (2003). "Unraveling the chemistry of chemokine receptor ligands." Chem Rev **103**(9): 3733-52.
Germain, R. N. (2002). "T-cell development and the CD4-CD8 lineage decision." Nat Rev Immunol **2**(5): 309-22.
Gett, A. V. and P. D. Hodgkin (2000). "A cellular calculus for signal integration by T cells." Nat Immunol **1**(3): 239-44.
Gordon, K. M., L. Duckett, et al. (2003). "A simple method for detecting up to five immunofluorescent parameters together with DNA staining for cell cycle or viability on a benchtop flow cytometer." J Immunol Methods **275**(1-2): 113-21.
Hanahan, D. (1998). "Peripheral-antigen-expressing cells in thymic medulla: factors in self-tolerance and autoimmunity." Curr Opin Immunol **10**(6): 656-62.
Hare, K. J., R. W. Wilkinson, et al. (1998). "Identification of a developmentally regulated phase of postselection expansion driven by thymic epithelium." J Immunol **160**(8): 3666-72.
Harman, B. C., E. J. Jenkinson, et al. (2003). "Entry into the thymic microenvironment triggers Notch activation in the earliest migrant T cell progenitors." J Immunol **170**(3): 1299-303.

Harman, B. C., E. J. Jenkinson, et al. (2003). "Microenvironmental regulation of Notch signalling in T cell development." Semin Immunol **15**(2): 91-7.

Hernandez-Lopez, C., A. Varas, et al. (2002). "Stromal cell-derived factor 1/CXCR4 signaling is critical for early human T-cell development." Blood **99**(2): 546-54.

Holler, P. D. and D. M. Kranz (2003). "Quantitative analysis of the contribution of TCR/pepMHC affinity and CD8 to T cell activation." Immunity **18**(2): 255-64.

Holmberg, K., S. Mariathasan, et al. (2003). "TCR binding kinetics measured with MHC class I tetramers reveal a positive selecting peptide with relatively high affinity for TCR." J Immunol **171**(5): 2427-34.

Hu, B., A. Punturieri, et al. (2002). "Recognition and phagocytosis of apoptotic T cells by resident murine tissue macrophages require multiple signal transduction events." J Leukoc Biol **71**(5): 881-9.

Hunig, T., N. Torres-Nagel, et al. (2001). "Thymic development and repertoire selection: the rat perspective." Immunol Rev **184**: 7-19.

Hutchinson, S. L., L. Wooldridge, et al. (2003). "The CD8 T cell coreceptor exhibits disproportionate biological activity at extremely low binding affinities." J Biol Chem **278**(27): 24285-93.

Imachi, H., K. Murao, et al. (2000). "Human scavenger receptor B1 is involved in recognition of apoptotic thymocytes by thymic nurse cells." Lab Invest **80**(2): 263-70.

Irvine, D. J., M. A. Purbhoo, et al. (2002). "Direct observation of ligand recognition by T cells." Nature **419**(6909): 845-9.

Ito, Y., S. Arai, et al. (2002). "Positive selection by the pre-TCR yields mature CD8+ T cells." J Immunol **169**(9): 4913-9.

Jacobsen, M., S. Cepok, et al. (2001). "New approaches to dissect degeneracy and specificity in T cell antigen recognition." J Mol Med **79**(7): 358-67.

Janeway, C. A., Jr. (1999). "T-cell development: a role for self-peptides in positive selection." Curr Biol **9**(9): R342-5.

Jin, C., W. X. Fu, et al. (2003). "SDF-1alpha production is negatively regulated by mouse estrogen enhanced transcript in a mouse thymus epithelial cell line." Cell Immunol **223**(1): 26-34.

Kangueane, P., M. K. Sakharkar, et al. (2001). "Towards the MHC-peptide combinatorics." Hum Immunol **62**(5): 539-56.

Kawamoto, H., K. Ohmura, et al. (2003). "Extensive proliferation of T cell lineage-restricted progenitors in the thymus: an essential process for clonal expression of diverse T cell receptor beta chains." Eur J Immunol **33**(3): 606-15.

Keller, R. (2001). "Dendritic cells: their significance in health and disease." Immunol Lett **78**(3): 113-22.

Kim, C. H. and H. E. Broxmeyer (1999). "SLC/exodus2/6Ckine/TCA4 induces chemotaxis of hematopoietic progenitor cells: differential activity of ligands of CCR7, CXCR3, or CXCR4 in chemotaxis vs. suppression of progenitor proliferation." J Leukoc Biol **66**(3): 455-61.

Kim, C. H., L. M. Pelus, et al. (1998). "Differential chemotactic behavior of developing T cells in response to thymic chemokines." Blood **91**(12): 4434-43.

King, L. B., E. Tolosa, et al. (1999). "A dominant-negative mutant of c-Jun inhibits cell cycle progression during the transition of CD4(-)CD8(-) to CD4(+)CD8(+) thymocytes." Int Immunol **11**(8): 1203-16.

Klug, D. B., C. Carter, et al. (1998). "Interdependence of cortical thymic epithelial cell differentiation and T-lineage commitment." Proc Natl Acad Sci U S A **95**(20): 11822-7.

Kremer, L., L. Carramolino, et al. (2001). "The transient expression of C-C chemokine receptor 8 in thymus identifies a thymocyte subset committed to become CD4+ single-positive T cells." J Immunol **166**(1): 218-25.

Kreuwel, H. T. and L. A. Sherman (2001). "The T-cell repertoire available for recognition of self-antigens." Curr Opin Immunol **13**(6): 639-43.

Kunkel, E. J. and E. C. Butcher (2002). "Chemokines and the tissue-specific migration of lymphocytes." Immunity **16**(1): 1-4.

Lee, K. H., A. R. Dinner, et al. (2003). "The immunological synapse balances T cell receptor signaling and degradation." Science **302**(5648): 1218-22.

Lee, W. H., T. Ramos, et al. (2003). "Development of T cells expressing an altered TCR complex." Eur J Immunol **33**(10): 2696-705.

Legrand, N. and A. A. Freitas (2001). "CD8+ T lymphocytes in double alpha beta TCR transgenic mice. I. TCR expression and thymus selection in the absence or in the presence of self-antigen." J Immunol **167**(11): 6150-7.

Lehar, S. M. and M. J. Bevan (2002). "T cell development in culture." Immunity **17**(6): 689-92.

Lepique, A. P., S. Palencia, et al. (2003). "Characterization of vascular adhesion molecules that may facilitate progenitor homing in the post-natal mouse thymus." Clin Dev Immunol **10**(1): 27-33.

Lilic, M., F. R. Santori, et al. (2002). "The role of fibroblasts in thymocyte-positive selection." J Immunol **169**(9): 4945-50.

Lind, E. F., S. E. Prockop, et al. (2001). "Mapping precursor movement through the postnatal thymus reveals specific microenvironments supporting defined stages of early lymphoid development." J Exp Med **194**(2): 127-34.

Lind, E. F., J. Wayne, et al. (1999). "Bcl-2-induced changes in E2F regulatory complexes reveal the potential for integrated cell cycle and cell death functions." J Immunol **162**(9): 5374-9.

Lipscomb, M. F. and B. J. Masten (2002). "Dendritic cells: immune regulators in health and disease." Physiol Rev **82**(1): 97-130.

Littman, D. R., Z. Sun, et al. (1999). "Role of the nuclear hormone receptor ROR gamma in transcriptional regulation, thymocyte survival, and lymphoid organogenesis." Cold Spring Harb Symp Quant Biol **64**: 373-81.

Liu, Y. J. (2001). "Dendritic cell subsets and lineages, and their functions in innate and adaptive immunity." Cell **106**(3): 259-62.

Livak, F., D. B. Burtrum, et al. (2000). "Genetic modulation of T cell receptor gene segment usage during somatic recombination." J Exp Med **192**(8): 1191-6.

Livak, F. and H. T. Petrie (2001). "Somatic generation of antigen-receptor diversity: a reprise." Trends Immunol **22**(11): 608-12.

Livak, F. and H. T. Petrie (2002). "Access roads for RAG-ged terrains: control of T cell receptor gene rearrangement at multiple levels." Semin Immunol **14**(5): 297-309.

Livak, F., H. T. Petrie, et al. (1995). "In-frame TCR delta gene rearrangements play a critical role in the alpha beta/gamma delta T cell lineage decision." Immunity **2**(6): 617-27.

Livak, F., M. Tourigny, et al. (1999). "Characterization of TCR gene rearrangements during adult murine T cell development." J Immunol **162**(5): 2575-80.

Lovatt, M., T. H. Yang, et al. (2000). "Different doses of agonistic ligand drive the maturation of functional CD4 and CD8 T cells from immature precursors." Eur J Immunol **30**(2): 371-81.
Love, P. E. and A. C. Chan (2003). "Regulation of thymocyte development: only the meek survive." Curr Opin Immunol **15**(2): 199-203.
Lucas, A. D. and D. R. Greaves (2001). "Atherosclerosis: role of chemokines and macrophages." Expert Rev Mol Med **3**: 1-18.
Luster, A. D. (1998). "Chemokines--chemotactic cytokines that mediate inflammation." N Engl J Med **338**(7): 436-45.
Lyons, D. S., S. A. Lieberman, et al. (1996). "A TCR binds to antagonist ligands with lower affinities and faster dissociation rates than to agonists." Immunity **5**(1): 53-61.
Mak, T. W., J. M. Penninger, et al. (2001). "Knockout mice: a paradigm shift in modern immunology." Nat Rev Immunol **1**(1): 11-9.
Malissen, B. (2003). "Immunology. Switching off TCR signaling." Science **302**(5648): 1162-3.
Mariathasan, S., A. Zakarian, et al. (2001). "Duration and strength of extracellular signal-regulated kinase signals are altered during positive versus negative thymocyte selection." J Immunol **167**(9): 4966-73.
Mazel, S., D. Burtrum, et al. (1996). "Regulation of cell division cycle progression by bcl-2 expression: a potential mechanism for inhibition of programmed cell death." J Exp Med **183**(5): 2219-26.
McHugh, R. S. and E. M. Shevach (2002). "Cutting edge: depletion of CD4+CD25+ regulatory T cells is necessary, but not sufficient, for induction of organ-specific autoimmune disease." J Immunol **168**(12): 5979-83.
Mellman, I. and R. M. Steinman (2001). "Dendritic cells: specialized and regulated antigen processing machines." Cell **106**(3): 255-8.
Michie, A. M. and J. C. Zuniga-Pflucker (2002). "Regulation of thymocyte differentiation: pre-TCR signals and beta-selection." Semin Immunol **14**(5): 311-23.
Miller, M. J., S. H. Wei, et al. (2002). "Two-photon imaging of lymphocyte motility and antigen response in intact lymph node." Science **296**(5574): 1869-73.
Mora, J. R., M. R. Bono, et al. (2003). "Selective imprinting of gut-homing T cells by Peyer's patch dendritic cells." Nature **424**(6944): 88-93.
Murgia, C., P. Blaikie, et al. (1998). "Cell cycle and adhesion defects in mice carrying a targeted deletion of the integrin beta4 cytoplasmic domain." Embo J **17**(14): 3940-51.
Murphy, P. M., M. Baggiolini, et al. (2000). "International union of pharmacology. XXII. Nomenclature for chemokine receptors." Pharmacol Rev **52**(1): 145-76.
Nabarra, B., M. Mulotte, et al. (2001). "Ultrastructural study of the FVB/N mouse thymus: presence of an immature epithelial cell in the medulla and premature involution." Dev Comp Immunol **25**(3): 231-43.
Nauta, A. J., M. R. Daha, et al. (2003). "Recognition and clearance of apoptotic cells: a role for complement and pentraxins." Trends Immunol **24**(3): 148-54.
Newman, M. (2000). "Applied mathematics. The power of design." Nature **405**(6785): 412-3.
Norment, A. M. and M. J. Bevan (2000). "Role of chemokines in thymocyte development." Semin Immunol **12**(5): 445-55.

Norment, A. M., L. Y. Bogatzki, et al. (2000). "Murine CCR9, a chemokine receptor for thymus-expressed chemokine that is up-regulated following pre-TCR signaling." J Immunol **164**(2): 639-48.
Ohashi, P. S. (2003). "Negative selection and autoimmunity." Curr Opin Immunol **15**(6): 668-76.
Oliveira-dos-Santos, A. J., T. Rieker-Geley, et al. (1997). "Murine thymic nurse cells and rosettes: analysis of adhesion molecule expression using confocal microscopy and a simplified enrichment method." J Histochem Cytochem **45**(9): 1293-7.
Oosterwegel, M. A., M. C. Haks, et al. (1997). "Induction of TCR gene rearrangements in uncommitted stem cells by a subset of IL-7 producing, MHC class-II-expressing thymic stromal cells." Immunity **6**(3): 351-60.
Palmer, E. (2003). "Negative selection--clearing out the bad apples from the T-cell repertoire." Nat Rev Immunol **3**(5): 383-91.
Penit, C., B. Lucas, et al. (1995). "Cell expansion and growth arrest phases during the transition from precursor (CD4-8-) to immature (CD4+8+) thymocytes in normal and genetically modified mice." J Immunol **154**(10): 5103-13.
Petrie, H. T. (2000). "Induction and control of thymic lymphopoiesis and homeostasis." Semin Immunol **12**(5): 419-420.
Petrie, H. T. (2002). "Role of thymic organ structure and stromal composition in steady-state postnatal T-cell production." Immunol Rev **189**: 8-19.
Petrie, H. T., F. Livak, et al. (1995). "T cell receptor gene recombination patterns and mechanisms: cell death, rescue, and T cell production." J Exp Med **182**(1): 121-7.
Petrie, H. T., F. Livak, et al. (1993). "Multiple rearrangements in T cell receptor alpha chain genes maximize the production of useful thymocytes." J Exp Med **178**(2): 615-22.
Petrie, H. T., A. Strasser, et al. (1993). "CD4+8- and CD4-8+ mature thymocytes require different post-selection processing for final development." J Immunol **151**(3): 1273-9.
Petrie, H. T., M. Tourigny, et al. (2000). "Precursor thymocyte proliferation and differentiation are controlled by signals unrelated to the pre-TCR." J Immunol **165**(6): 3094-8.
Petrie, H. T. and W. Van Ewijk (2002). "Thymus by numbers." Nat Immunol **3**(7): 604-5.
Petrie-Hanson, L. and A. J. Ainsworth (2000). "Differential cytochemical staining characteristics of channel catfish leukocytes identify cell populations in lymphoid organs." Vet Immunol Immunopathol **73**(2): 129-44.
Petrie-Hanson, L. and A. J. Ainsworth (2001). "Ontogeny of channel catfish lymphoid organs." Vet Immunol Immunopathol **81**(1-2): 113-27.
Pezzano, M., M. Samms, et al. (2001). "Questionable thymic nurse cell." Microbiol Mol Biol Rev **65**(3): 390-403, table of contents.
Pitkanen, J. and P. Peterson (2003). "Autoimmune regulator: from loss of function to autoimmunity." Genes Immun **4**(1): 12-21.
Platt, N., H. Suzuki, et al. (2000). "Apoptotic thymocyte clearance in scavenger receptor class A-deficient mice is apparently normal." J Immunol **164**(9): 4861-7.
Platt, N., H. Suzuki, et al. (1996). "Role for the class A macrophage scavenger receptor in the phagocytosis of apoptotic thymocytes in vitro." Proc Natl Acad Sci U S A **93**(22): 12456-60.

Plotkin, J., S. E. Prockop, et al. (2003). "Critical role for CXCR4 signaling in progenitor localization and T cell differentiation in the postnatal thymus." J Immunol **171**(9): 4521-7.
Porritt, H. E., K. Gordon, et al. (2003). "Kinetics of steady-state differentiation and mapping of intrathymic-signaling environments by stem cell transplantation in nonirradiated mice." J Exp Med **198**(6): 957-62.
Powrie, F. and K. J. Maloy (2003). "Immunology. Regulating the regulators." Science **299**(5609): 1030-1.
Poznansky, M. C., I. T. Olszak, et al. (2002). "Thymocyte emigration is mediated by active movement away from stroma-derived factors." J Clin Invest **109**(8): 1101-10.
Prockop, S. and H. T. Petrie (2000). "Cell migration and the anatomic control of thymocyte precursor differentiation." Semin Immunol **12**(5): 435-44.
Prockop, S. E., S. Palencia, et al. (2002). "Stromal cells provide the matrix for migration of early lymphoid progenitors through the thymic cortex." J Immunol **169**(8): 4354-61.
Ramialison, M., E. Mohr, et al. (2002). "Expression profiling in mouse fetal thymus reveals clusters of coordinately expressed genes that mark individual stages of T-cell ontogeny." Immunogenetics **54**(7): 469-78.
Robey, E. and M. Schlissel (2003). "Lymphocyte development." Curr Opin Immunol **15**(2): 155-7.
Romagnani, P., F. Annunziato, et al. (2001). "Interferon-inducible protein 10, monokine induced by interferon gamma, and interferon-inducible T-cell alpha chemoattractant are produced by thymic epithelial cells and attract T-cell receptor (TCR) alphabeta+ CD8+ single-positive T cells, TCRgammadelta+ T cells, and natural killer-type cells in human thymus." Blood **97**(3): 601-7.
Rossi, D. and A. Zlotnik (2000). "The biology of chemokines and their receptors." Annu Rev Immunol **18**: 217-42.
Samms, M., M. Martinez, et al. (2001). "Circulating macrophages as well as developing thymocytes are enclosed within thymic nurse cells." Cell Immunol **212**(1): 16-23.
Samms, M., D. Philp, et al. (1999). "Lysosomal-mediated degradation of apoptotic thymocytes within thymic nurse cells." Cell Immunol **197**(2): 108-15.
Savino, W., S. Ayres Martins, et al. (2003). "Thymocyte migration: an affair of multiple cellular interactions?" Braz J Med Biol Res **36**(8): 1015-25.
Savino, W., D. A. Mendes-da-Cruz, et al. (2002). "Intrathymic T-cell migration: a combinatorial interplay of extracellular matrix and chemokines?" Trends Immunol **23**(6): 305-13.
Schmeissner, P. J., H. Xie, et al. (2001). "Integrin functions play a key role in the differentiation of thymocytes in vivo." J Immunol **167**(7): 3715-24.
Schuurman, H. J., C. F. Kuper, et al. (1997). "Thymic microenvironment at the light microscopic level." Microsc Res Tech **38**(3): 216-26.
Schwartz, M. and J. Kipnis (2002). "Autoimmunity on alert: naturally occurring regulatory CD4(+)CD25(+) T cells as part of the evolutionary compromise between a 'need' and a 'risk'." Trends Immunol **23**(11): 530-4.
Scollay, R. and D. I. Godfrey (1995). "Thymic emigration: conveyor belts or lucky dips?" Immunol Today **16**(6): 268-73; discussion 273-4.
Sebzda, E., S. Mariathasan, et al. (1999). "Selection of the T cell repertoire." Annu Rev Immunol **17**: 829-74.

Seddon, B. and D. Mason (2000). "The third function of the thymus." Immunol Today **21**(2): 95-9.
Sercarz, E. E. and E. Maverakis (2003). "Mhc-guided processing: binding of large antigen fragments." Nat Rev Immunol **3**(8): 621-9.
Shevach, E. M. (2002). "CD4+ CD25+ suppressor T cells: more questions than answers." Nat Rev Immunol **2**(6): 389-400.
Sohn, S. J., A. Rajpal, et al. (2003). "Apoptosis during lymphoid development." Curr Opin Immunol **15**(2): 209-16.
Souto, P. C., V. N. Brito, et al. (2003). "Programmed cell death in thymus during experimental paracoccidioidomycosis." Med Microbiol Immunol (Berl) **192**(4): 225-9.
Spits, H. (2002). "Development of alphabeta T cells in the human thymus." Nat Rev Immunol **2**(10): 760-72.
Sprent, J. and H. Kishimoto (2002). "The thymus and negative selection." Immunol Rev **185**: 126-35.
Starr, T. K., S. C. Jameson, et al. (2003). "Positive and negative selection of T cells." Annu Rev Immunol **21**: 139-76.
Stephens, G. L. and L. Ignatowicz (2003). "Decreasing the threshold for thymocyte activation biases CD4+ T cells toward a regulatory (CD4+CD25+) lineage." Eur J Immunol **33**(5): 1282-91.
Stoll, S., J. Delon, et al. (2002). "Dynamic imaging of T cell-dendritic cell interactions in lymph nodes." Science **296**(5574): 1873-6.
Suzuki, G., Y. Nakata, et al. (1998). "Loss of SDF-1 receptor expression during positive selection in the thymus." Int Immunol **10**(8): 1049-56.
Suzuki, G., H. Sawa, et al. (1999). "Pertussis toxin-sensitive signal controls the trafficking of thymocytes across the corticomedullary junction in the thymus." J Immunol **162**(10): 5981-5.
Taylor, J. R., Jr., K. C. Kimbrell, et al. (2001). "Expression and function of chemokine receptors on human thymocytes: implications for infection by human immunodeficiency virus type 1." J Virol **75**(18): 8752-60.
Terpstra, V., N. Kondratenko, et al. (1997). "Macrophages lacking scavenger receptor A show a decrease in binding and uptake of acetylated low-density lipoprotein and of apoptotic thymocytes, but not of oxidatively damaged red blood cells." Proc Natl Acad Sci U S A **94**(15): 8127-31.
Teshima, T., P. Reddy, et al. (2003). "Impaired thymic negative selection causes autoimmune graft-versus-host disease." Blood **102**(2): 429-35.
Tourigny, M. R., S. Mazel, et al. (1997). "T cell receptor (TCR)-beta gene recombination: dissociation from cell cycle regulation and developmental progression during T cell ontogeny." J Exp Med **185**(9): 1549-56.
Uehara, S., A. Grinberg, et al. (2002). "A role for CCR9 in T lymphocyte development and migration." J Immunol **168**(6): 2811-9.
Uehara, S., K. Song, et al. (2002). "Characterization of CCR9 expression and CCL25/thymus-expressed chemokine responsiveness during T cell development: CD3(high)CD69+ thymocytes and gammadeltaTCR+ thymocytes preferentially respond to CCL25." J Immunol **168**(1): 134-42.
Ueno, T., K. Hara, et al. (2002). "Role for CCR7 ligands in the emigration of newly generated T lymphocytes from the neonatal thymus." Immunity **16**(2): 205-18.
van der Merwe, P. A. and S. J. Davis (2003). "Molecular interactions mediating T cell antigen recognition." Annu Rev Immunol **21**: 659-84.

van Lent, P. L., R. Licht, et al. (2001). "Uptake of apoptotic leukocytes by synovial lining macrophages inhibits immune complex-mediated arthritis." J Leukoc Biol **70**(5): 708-14.

Vanhecke, D., B. Verhasselt, et al. (1997). "Human thymocytes become lineage committed at an early postselection CD69+ stage, before the onset of functional maturation." J Immunol **159**(12): 5973-83.

Viret, C., X. He, et al. (2003). "Altered positive selection due to corecognition of floppy peptide/MHC II conformers supports an integrative model of thymic selection." Proc Natl Acad Sci U S A **100**(9): 5354-9.

von Andrian, U. H. and C. R. Mackay (2000). "T-cell function and migration. Two sides of the same coin." N Engl J Med **343**(14): 1020-34.

von Boehmer, H., I. Aifantis, et al. (2003). "Thymic selection revisited: how essential is it?" Immunol Rev **191**: 62-78.

von Greyerz, S., G. Bultemann, et al. (2001). "Degeneracy and additional alloreactivity of drug-specific human alpha beta(+) T cell clones." Int Immunol **13**(7): 877-85.

Vukmanovic, S., T. A. Neubert, et al. (2003). "Could TCR antagonism explain associations between MHC genes and disease?" Trends Mol Med **9**(4): 139-46.

Wang, J. and M. J. Eck (2003). "Assembling atomic resolution views of the immunological synapse." Curr Opin Immunol **15**(3): 286-93.

Wang, R., Y. Wang-Zhu, et al. (2002). "Interactions between double positive thymocytes and high affinity ligands presented by cortical epithelial cells generate double negative thymocytes with T cell regulatory activity." Proc Natl Acad Sci U S A **99**(4): 2181-6.

Wayne, J., H. Suh, et al. (1994). "TCR selection and allelic exclusion in RAG transgenic mice that exhibit abnormal T cell localization in lymph nodes and lymphatics." J Immunol **153**(12): 5491-502.

Wilkinson, R. W., G. Anderson, et al. (1995). "Positive selection of thymocytes involves sustained interactions with the thymic microenvironment." J Immunol **155**(11): 5234-40.

Wilson, D. B., C. Pinilla, et al. (1999). "Immunogenicity. I. Use of peptide libraries to identify epitopes that activate clonotypic CD4+ T cells and induce T cell responses to native peptide ligands." J Immunol **163**(12): 6424-34.

Wurbel, M. A., M. Malissen, et al. (2001). "Mice lacking the CCR9 CC-chemokine receptor show a mild impairment of early T- and B-cell development and a reduction in T-cell receptor gammadelta(+) gut intraepithelial lymphocytes." Blood **98**(9): 2626-32.

Wurbel, M. A., J. M. Philippe, et al. (2000). "The chemokine TECK is expressed by thymic and intestinal epithelial cells and attracts double- and single-positive thymocytes expressing the TECK receptor CCR9." Eur J Immunol **30**(1): 262-71.

Yannoutsos, N., P. Wilson, et al. (2001). "The role of recombination activating gene (RAG) reinduction in thymocyte development in vivo." J Exp Med **194**(4): 471-80.

Youn, B. S., C. H. Kim, et al. (1999). "TECK, an efficacious chemoattractant for human thymocytes, uses GPR-9-6/CCR9 as a specific receptor." Blood **94**(7): 2533-6.

Yu, Q., B. Erman, et al. (2003). "In vitro evidence that cytokine receptor signals are required for differentiation of double positive thymocytes into functionally mature CD8+ T cells." J Exp Med **197**(4): 475-87.
Zaballos, A., J. Gutierrez, et al. (1999). "Cutting edge: identification of the orphan chemokine receptor GPR-9-6 as CCR9, the receptor for the chemokine TECK." J Immunol **162**(10): 5671-5.
Zabel, B. A., W. W. Agace, et al. (1999). "Human G protein-coupled receptor GPR-9-6/CC chemokine receptor 9 is selectively expressed on intestinal homing T lymphocytes, mucosal lymphocytes, and thymocytes and is required for thymus-expressed chemokine-mediated chemotaxis." J Exp Med **190**(9): 1241-56.
Zaitseva, M., T. Kawamura, et al. (2002). "Stromal-derived factor 1 expression in the human thymus." J Immunol **168**(6): 2609-17.
Zaitseva, M. B., S. Lee, et al. (1998). "CXCR4 and CCR5 on human thymocytes: biological function and role in HIV-1 infection." J Immunol **161**(6): 3103-13.
Zhao, Y., B. Gran, et al. (2001). "Combinatorial peptide libraries and biometric score matrices permit the quantitative analysis of specific and degenerate interactions between clonotypic TCR and MHC peptide ligands." J Immunol **167**(4): 2130-41.
Zinkernagel, R. M. and A. Althage (1999). "On the role of thymic epithelium vs. bone marrow-derived cells in repertoire selection of T cells." Proc Natl Acad Sci U S A **96**(14): 8092-7.
Zippelius, A., M. J. Pittet, et al. (2002). "Thymic selection generates a large T cell pool recognizing a self-peptide in humans." J Exp Med **195**(4): 485-94.
Zlotnik, A. and O. Yoshie (2000). "Chemokines: a new classification system and their role in immunity." Immunity **12**(2): 121-7.
